# Seroprevalence of Hepatitis B virus infection and associated factors among pregnant women at Korle-Bu Teaching Hospital, Ghana

**DOI:** 10.1371/journal.pone.0232208

**Published:** 2020-04-22

**Authors:** Benjamin Ansah Dortey, Emmanuel Anongeba Anaba, A. T. Lassey, N. K. R. Damale, Ernest T. Maya

**Affiliations:** 1 Trauma and Specialist Hospital, Winneba, Ghana; 2 Department of Population, Family and Reproductive Health, University of Ghana School of Public Health, Accra, Ghana; 3 University of Ghana School of Medicine and Dentistry, Accra, Ghana; Centre de Recherche en Cancerologie de Lyon, FRANCE

## Abstract

**Introduction:**

Hepatitis B virus infection is a global public health problem. Though, the disease is endemic in sub-Saharan Africa, little is known about its epidemiology among pregnant women in Ghana. This study sought to determine the seroprevalence of Hepatitis B virus infection and associated factors among pregnant women attending antenatal care at Korle-Bu Teaching Hospital; Ghana’s largest hospital.

**Methods:**

We conducted a facility-based cross-sectional survey among 232 antenatal attendants. Participants were recruited using systematic random sampling technique and screened with HBsAg Rapid Test. Data was analyzed with the aid of Statistical Package for Social Sciences (SPSS), version 23.0. Results were presented using descriptive statistics, Fisher’s Exact test and Logistic Regression analysis.

**Results:**

Two hundred and twenty-one (221) of the total sample (n = 232) agreed to participate in this study; representing a response rate of 95%. The mean age of the participants was 31 years and standard deviation of 5.3. The mean gestational period at recruitment was 28 weeks and standard deviation of 6.8. Majority of the participants were married (83.3%), parous (69.6%), educated (91.4%) and employed (90.5%). The prevalence of HBsAg was 7.7%. We found no significant association between socio-demographic characteristics of the participants and HBV infection.

**Conclusion:**

Seroprevalence of 7.7% indicates moderate endemicity. Socio-demographic characteristics did not influence HBV infection among pregnant women attending antenatal care at Korle-Bu Teaching Hospital. The findings provide empirical evidence that will contribute to knowledge of HBV epidemiology in Ghana.

## Introduction

Hepatitis B Virus (HBV) infection is a global public health problem. Over 380 million people worldwide are chronic carriers of HBV and over two million deaths occur annually from HBV-related diseases [1]. Persons with HBV infection are at higher risk of liver cirrhosis and hepatocellular carcinoma (HCC) [1, 2]. The global prevalence of HBV infection varies widely from high (≥ 8%) in Africa, Asia and the Western Pacific to moderate (2–7%) in Southern and Eastern Europe and low (< 2%) in Western Europe, North America and Australia [3]. HBV is the commonest hepatitis virus during pregnancy [2]. Evidence show that the prevalence of HBV infection among pregnant women in sub-Saharan Africa ranges between 2.4% in Ethiopia to 11.8% in Uganda [4–7]. Prior studies on HBV infection among pregnant women in Ghana found a prevalence of 7.9% and 9.5% in the middle and northern parts respectively [8, 9].

Some studies have shown that there is a significant relationship between participants’ characteristics and HBV infection during pregnancy. For example, a retrospective study in Cameroon found a significant relationship between occupation [10] and age [6, 11], and HBV infection among pregnant women [11]. In Nigeria, Anaedobe, Fowotade [12], revealed that pregnant women with multiple sexual partners [6, 10, 13–15] and those who experienced early sexual debut were more likely to be infected with HBV. Other risk factors include marital status, history of abortion, blood transfusion and surgery, family history of HBV infection, and alcohol intake [10–16]. However, other studies in sub-Saharan Africa including Ghana, found no significant association between participants’ characteristics and HBV positivity [8, 9].

Acute Hepatitis B infection during pregnancy can cause premature delivery [17, 18]. Perinatal transmission is the commonest mode of HBV transmission worldwide, contributing close to 40% of all chronic HBV infections in sub-Saharan Africa [19, 20]. Infants born to a seropositive mother for both HBsAg and HBeAg have 70–90% chance of acquiring perinatal HBV infection, while those born to seropositive mothers with HBsAg but negative to HBeAg have 25% chance of acquiring perinatal HBV infection. Infants who are infected perinatally have 85–90% chance of becoming chronic HBV carriers and can propagate horizontal transmission. It has been estimated that more than 25% of these carriers will die of hepatocellular carcinoma (HCC) or cirrhosis of the liver [3, 21].

In Ghana, there is scanty national data on HBV [22]. However, hospital-based data indicate an increasing trend across the ten regions of Ghana [23]. Statistics available indicate that about four million Ghanaians are living with Hepatitis B or C, with majority being unaware of their status [22]. In addition, empirical literature on HBV epidemiology in Ghana, especially among pregnant women, is limited. The few existing studies are skewed toward the middle and northern parts of Ghana [8, 9, 16]. Moreover, considering the grave consequences of the diseases on pregnancy outcomes and infants, it is importance to explore more risk factors associated with the disease. Understanding the current prevalence and risk factors can inform health policies and interventions that can help improve pregnancy outcomes, and also reduce the risk of perinatal transmission. With the increasing public awareness and vaccination against HBV among the Ghanaian populace, it would be necessary to know the current prevalence of the disease for healthcare planning. The findings of this study would also contribute to the scanty literature on the disease. Therefore, this study sought to determine HBsAg seroprevalence and associated factors among pregnant women attending antenatal care at Korle-Bu Teaching Hospital (KBTH) in southern Ghana.

## Methods

### Research design and setting

A cross-sectional survey was conducted at the Obstetrics and Gynaecology department of the KBTH. It was established in 1923 and represents Ghana’s premier teaching hospital with a total bed capacity of 2,000. The hospital attends mostly to cases referred from lower level health facilities. According to the 2017 Ghana Maternal Health Survey report, the national antenatal coverage for women in reproductive age (15–49) is 98%, with 89% making the recommended 4+ ANC visits [24]. The Greater Accra Region; the study setting, has a general coverage of 97.5% and 93.0% coverage for the recommended 4+ANC visits, which is the second highest after the Upper East Region [24]. In Ghana, HBV screening is part of routine antenatal care. However, national data on HBV vaccination coverage, especially among pregnant women is limited. Ghana developed a national policy on viral hepatitis not long ago (2014) [22]. The policy seeks to provide directions and guidelines for surveillance, prevention and control, and treatment of viral hepatitis. The implementation of the policy, however, has been problematic.

### Study population

The target population for this study was all pregnant women attending the ANC clinic at KBTH. The study sample was selected from the booking ANC clinic. Apart from the fact that the study population was representative of the clientele at the hospital, it also avoided duplication of data, as clients attend this clinic only once. The clinic has an average daily attendance of twenty-five clients.

### Sample size and sampling

A sample size of 232 pregnant women was calculated based on 0.05 margin of error, 95% confidence level and 10.5% seroprevalence from a previous study [17]. Study participants were recruited using systematic random sampling technique. Using the attendance book at the booking clinic, the first participant was randomly selected from the first three attendants by balloting. The next participant was the third attendant after the first attendant sampled and then it followed. If an attendant declined to participate, the third attendant after her was selected.

### Study procedures

Participants were recruited from the booking clinic by the first author (BAD). Trained research assistants approached potential participants and explained the purpose of the study to them. Participants who were interested were taken through a comprehensive informed consent process. A structured questionnaire was used to collect participants socio-demographic data through face-to-face interviews using either English and the predominant local languages (i.e. Ga and Twi).

#### Screening for HBsAg among pregnant women attending antenatal care at KBTH

After administering the questionnaire and with participants permission, three milliliters(ml) of a participant’s blood was collected by venipuncture into EDTA tubes and allowed to stand for 30 minutes. This separated the plasma and the red blood cells. The Onsite HBsAg Rapid Test (CTK Biotech. Inc, San Diego, CA 92121, USA) was used for the HBsAg test. The kit consists of one dip strip device, a plastic pipette and one desiccant. The Onsite Rapid HBsAg Test is a lateral flow chromatographic immunoassay. It qualitatively detects HBsAg in human serum or plasma at concentrations greater than or equal to 1ng/ml. It has a relative sensitivity of 100% and a relative specificity of 100% when compared to the HBsAg ELISA kit test (confirmatory test) [25]. A plastic pipette was used to draw about 0.5ml of the plasma and one drop was put unto the Onsite Rapid HBsAg Test cassette. One drop of the diluent was immediately added to the drop of plasma in the cassette and the result was read in 15 minutes. A test was classified as positive for HBsAg if both the control (C) and test (T) bands on the test cassette developed and negative if only the C band developed and invalid if the C band did not develop. All invalid tests were repeated. The screening was performed by a biomedical scientist at KBTH.

### Ethical consideration

Ethical approval to carry out the study was obtained from the Ethical and Protocol Review Committee of the College of Health Sciences of the University of Ghana (MS-Et/M.3- P4.3/ 2015–2016). Permission was obtained from the Department of Obstetrics and Gynecology, KBTH to carry out the study in the department. Written informed consent was obtained from all study participants. Participants were informed that participation was voluntary and they were at liberty to withdraw from the study at any time without any consequences to them. They were told 3ml of their blood would be drawn for the HBsAg testing and that they may experience minimal pain which is no different from the pain they experience when their blood is drawn for other antenatal tests. The participants were assured of confidentiality of the test results and any information obtained from them. They were told their names would not appear in any presentation or publication of the study findings. They were also made aware that the test cassette would be stored for a period of two years before disposal. All study procedures were conducted on one-to-one basis in a private room at the antenatal clinic. All the questionnaires and the Onsite HBsAg Rapid test kit were kept in a locked cabinet accessible to only the principal investigator. The soft copies of the de-identified data were stored on a password protected personal computer of the principal investigator.

### Data analysis

The questionnaire was coded into Statistical Package for Social Sciences (SPSS) software, version 23.0. Data entry was done by the principal investigator. The data were cross-checked for wrong and omitted entries by computing descriptive statistics for all the variables. Normality of the data was checked using Shapiro-Wilk test. The main outcome (dependent variable) in this study was HBsAg status, which was as coded as 1 = ‘positive’ and 0 = ‘negative’. Independent variables identified in literature included age, educational status, marital status, employment status, parity, gestational age and partner’s employment status. Age, parity and gestational period were continuous variables. Descriptive statistics were computed using frequency, percentage, mean and standard deviation. Fisher’s Exact test was also computed for association between HBsAg status and participants’ characteristics. In addition, Logistic Regression analysis was computed to determine predictors of HBsAg status. Assumptions underlying Fisher’s Exact test and Logistic regression, such as outliers and multicollinearity were all satisfied.

## Results

### Socio-demographic characteristics of study participants

Of the 232 pregnant women who were invited to participate in the study, 221 honored the invitation, representing 95% response rate. The mean age of the participants was 31 (SD ± 5.3) years. Participants who were above age 30 constituted about half (49.8%) of the total participants. About eight in ten participants were married, while, nine in ten participants had at least primary education. Majority (69.6%) of the participants were parous and 54.8% of the participants were in their second trimester. The mean gestational age (at recruitment was 28.0 (SD ± 6.8) weeks. Majority of the participants (90.5%) and their partners were employed. Details are provided in **Table 1**

**Table 1:** Descriptive statistics on socio-demographic characteristics of pregnant women attending antenatal care at Korle-Bu Teaching Hospital, Ghana (n = 221).

Socio-demographic characteristics	Frequency (n)	Percentage (%)
**Age (years)**	Mean (SD ±) 31(5.3)	
18–24	31	14.0
25–30	80	36.2
31–43	110	49.8
**Educational level**		
No formal education	19	8.6
Primary	14	6.3
Junior High	66	29.9
Senior High	73	33.0
Tertiary	49	22.2
**Marital status**		
Married	184	83.3
Single	37	16.7
**Employment status**		
Unemployed	21	9.5
Informal sector employee	148	67.0
Formal sector employee	52	23.5
**Parity**	Mean (SD ±) 1(1.3)	
Nulliparous	67	30.4
Primiparous	77	34.8
Multiparous	77	34.8
**Gestation period (weeks)**	Mean (SD ±) 28(6.8)	
First trimester	0	0
Second trimester	121	54.8
Third trimester	100	45.2
**Partner’s employment status**		
Unemployed	0	0
Informal sector employee	143	65.0
Formal sector employee	77	35.0

### Association between socio-demographic characteristics and HBV infection

A total of 17 (7.7%) of the participants tested positive for HBV infection. The age group with the highest prevalence was 25–30 years (11.3%). The highest HBV prevalence was found among participants with no formal education (10.5%). Regarding employment status, the highest prevalence was found among unemployed participants (14.3%). It was also found that most of the seropositive participants were multiparous (13.2%). We found no statistically significant association between socio-demographic characteristics of participants and HBsAg status. Details are provided in **Table 2**.

**Table 2:** Fisher’s Exact test on socio-demographic characteristics and HBsAg status among pregnant women attending antenatal care at Korle-Bu Teaching Hospital, Ghana (n = 221).

Participant’s characteristics	n	HBsAg status		Fisher’s Exact test	p-value
		Positive n (%)	Negative n (%)		
**Age (years)**					
18–24	31	2(6.5)	29(93.5)	2.116	.36
25–30	80	9(11.3)	71(88.7)		
31–43	110	6(5.5)	104(94.5)		
**Educational level**					
No formal education	19	2(10.5)	17(89.5)		
Primary	14	1(7.1)	13(92.9)	1.057	.93
Junior High	66	6(9.2)	60(90.8)		
Senior High	73	5(6.8)	68(93.2)		
Tertiary	49	3(6.1)	46(93.9)		
**Marital status**					
Married	184	15(8.2)	169(91.8)	.336	.74
Single	37	2(5.4)	35(94.6)		
**Employment status**					
Unemployed	21	3(14.3)	18(85.7)		
Informal sector employee	148	12(8.2)	135(91.8)	.248	.24
Formal sector employee	52	2(3.8)	50(96.2)		
**Parity**					
Nulliparous	67	4(6.0)	63(94.0)		
Primiparous	77	3(3.9)	74(96.1)	4.556	.10
Multiparous	77	10(13.2)	67(86.8)		
**Gestation period**					
First trimester	0	0	0		
Second trimester	121	11(9.1)	110(90.9)	.701	.46
Third trimester	100	6(6.1)	94(93.9)		
**Partner’s employment status**					
Unemployed	0	0	0		
Informal sector employee	143	12(8.5)	131(91.5)	.267	.79
Formal sector employee	77	5(6.5)	72(93.5)		

### Risk factors of HBV infection among pregnant women attending antenatal care at KBTH

At the multivariate level, the logistic regression model contained seven independent variables (age, educational status, marital status, employment status, parity, gestational period and partner’s employment status). The full model containing all predictors was not statistically significant, χ^2^ (7, n = 221) = 7.962, *p* > 0.05, indicating that the model was not able to distinguish between participants who tested negative and participants who tested positive. The model as a whole explained between 3.6% and 8.5% of the variance in HBV infection status and correctly classified 92.2% of cases. As shown in Table 3, none of the independent variables made a unique statistically significant contribution to the model (age, educational level, marital status, employment status, parity, gestational period and partner’s employment status). This indicates that none of the independent variables was a risk factor of HBV infection among pregnant women attending antenatal care at Korle-Bu Teaching Hospital.

**Table 3:** Logistic regression on socio-demographic characteristics and HBsAg status among pregnant women attending antenatal care at Korle-Bu Teaching Hospital, Ghana (n = 221).

Predictors	B	S.E.	Wald	df	p-value	Odd Ratio	95% C.I. for Odd Ratio	
							Lower	Upper
Age (years)	-.083	.063	1.723	1	.19	.921	.814	1.042
Education	.049	.252	.038	1	.85	1.050	.641	1.721
Marital status	-.833	.834	.997	1	.32	.435	.085	2.229
Employment status	-.937	.541	2.999	1	.08	.392	.136	1.131
Parity	.383	.242	2.498	1	.11	1.467	.912	2.360
Gestational period	-.061	.042	2.063	1	.15	.941	.866	1.022
Partner’s employment status	-.002	.617	.000	1	.99	.998	.298	3.345
Constant	3.835	2.99	1.643	1	.20	46.32		

## Discussion

Ghana is found in one of the regions with the highest HBV prevalence. However, literature on the epidemiology of the disease among pregnant women is limited. This study, therefore, sought to determine the seroprevalence of HBV and associated factors among pregnant women attending antenatal care at Korle-Bu Teaching Hospital. The seroprevalence of HBV infection was 7.7%. Per World Health Organization’s criteria for HBV severity, (≥ 8%; high, 2–7%; moderate and < 2%; low), the prevalence of 7.7% indicates moderate endemicity. The age group with the highest prevalence was 25–30 years. Also, HBV positivity was higher among women without formal education, unemployed and multiparous. However, there was no significant association between socio-demographic characteristics of participants and HBV infection.

The findings of this study are similar to findings of previous studies. For example, a recent study of HBV infection among pregnant women in northern Ghana found a prevalence of 7.5% [9]. Another study in the middle zone of Ghana found a prevalence of 9.5% among antenatal attendants [8]. These findings suggest that HBV still is a public health problem in Ghana and poses a serious threat to all pregnant women, irrespective of their region of residence. The prevalence found in this study is lower than prevalence from in rural settings. A cross-sectional survey among pregnant women in rural Ghana found a seroprevalence of 16.7% [26]. This indicates that HBV prevalence is higher in rural Ghana [23].The differences in findings might be due to differences in awareness and access to vaccination centers.

In addition, HBV prevalence in Ghana appears to be declining over time. For instance, studies conducted between 1995–2002 recorded the highest prevalence of 17.3%, followed by 14.7% for studies between 2003–2009,10.2% for studies between 2010–2015 [27], and 7.7% in this study. These findings suggest that awareness of HBV is increasing among the populace or probably many Ghanaians are vaccinating against the HBV. Moreover, the decline in prevalence might be due to differences in sample sizes, HBV screening methods or variations in risky socio-cultural and behavioral practices across generations.

The above findings are also similar to studies conducted in other sub-Saharan Africa countries. For example, in the Gambia, a prevalence of 9.2% was found among pregnant women attending antenatal care in a referral hospital [28]. Anaedobe, Fowotade [12] found a prevalence of 8.3% % among pregnant women in the south western part of Nigeria, and among pregnant women in Uganda, a prevalence of 11.8% was found [6]. Though, the prevalence in this study is lower than the aforementioned countries, it is higher than the prevalence in Rwanda (3.1%) [4], Ethiopia (4.7%) [7] and Tanzania (5.2%) [14]. The differences in findings across countries within the subregion might be due to geographical variations, differences in screening and sampling methods and cultural and behavioral differences regarding risk factors.

Furthermore, we found no significant association between socio-demographic characteristics of participants and HBV infection. These findings are supported by prior studies in Ghana. For example, in the Northern Region, Anabire, Aryee [9] found that socio-demographic factors were not risk factors of HBV infection among pregnant women. Ephraim, Donko [8] also substantiated that socio-demographic factors did not influence HBV infection among pregnant women in the Ashanti Region. On the contrary, similar studies in Ethiopia, Uganda and Cameroon found significant association between socio-demographic factors, such as marital status, age and occupation and HBV infection among pregnant women [6, 10, 11]. These findings suggest that risk factors of HBV differ from country to country. Therefore, the differences in findings may be due to variations in contextual factors. It may also be explained by the fact that knowledge and awareness of HBV is generally low among the Ghanaian population, irrespective of social class.

The moderate endemicity of HBV among pregnant women suggest that infections could occur in infants through perinatal transmission. It is therefore recommended that all pregnant women attending ANC, irrespective of their socio-demographic characteristics, should be routinely screened for HBV infection. This would help in early detection of HBV infection among pregnant women. Hence, necessary interventions can be implemented, such as counselling seropositive women and educating them on the need for vaccinating their newborns against HBV. In addition, a national policy to vaccinate all pregnant women who test negative for HBV must be adopted so as to reduce the risk of mother-to-child transmission within the population.

## Limitations

Our study has some limitations. First, the sample size is too small for generalizations. Also, the small number of seropositive hepatitis B patients made it difficult to establish associations. In addition, our inability to employ ELIZA for sero-analysis might have accounted for the small number of seropositive cases. Notwithstanding, the screening method used in this study (i.e. immunochromatographic) has a relative sensitivity and specificity of 100% when compared to the confirmatory test and therefore can give accurate results. Moreover, the same screening method was adopted by a previous study [8]. Another limitation of this study is that some possible confounding factors, such as sexual behaviours, medical and family history, were not controlled. Our study was skewed towards socio-demographic determinants of HBV infection. Therefore, interpretation or generalization of the findings must take this limitation into consideration. It is therefore recommended that future studies should explore other possible risk factors of HBV infection.

## Conclusion

Seroprevalence of 7.7% indicates moderate endemicity. Socio-demographic characteristics did not influence HBV infection among pregnant women attending antenatal care at Korle-Bu Teaching Hospital. The interpretation of the findings must be done with caution due to some limitations of the study. Notwithstanding, the findings of this study provide empirical evidence that will contribute to knowledge of HBV epidemiology in Ghana, and can also stimulate further research on the disease.

## References

[1] Frambo A.A.B., et al Prevalence of HBsAg and knowledge about hepatitis B in pregnancy in the Buea Health District, Cameroon: a cross-sectional study. BMC research notes, 2014 7(1): 394–01. 10.1186/1756-0500-7-394 24965844 PMC4082373

[2] Wah C.P., et al Awareness and knowledge of hepatitis B infection and prevention and the use of hepatitis B vaccination in the Hong Kong adult Chinese population. Chinese Medical Journal, 2012 125(3): 422–27. 10.3760/cma.j.issn.0366-6999.2012.03.004 22490396

[3] Maddrey W.C. Hepatitis B: an important public health issue. Journal of medical virology, 2000 61(3): p. 362–366. 10.1002/1096-9071(200007)61:3<362::aid-jmv14>3.0.co;2-i 10861647

[4] Nyamusi M.M., Marete O.T., and Waweru W.R. Seroprevalence of hepatitis B among pregnant women in Kigali, Rwanda. International Journal of Community Medicine and Public Health, 2016 3(11): 3096–01. 10.18203/2394-6040.ijcmph20163918

[5] Dabsu R. and Ejeta E., Seroepidemiology of Hepatitis B and C Virus Infections among Pregnant Women Attending Antenatal Clinic in Selected Health Facilities in East Wollega Zone, West Oromia, Ethiopia. BioMed research international, 2018 10.1155/2018/4792584 PMC6311240 30643809

[6] Bayo P., et al High prevalence of hepatitis B virus infection among pregnant women attending antenatal care: a cross-sectional study in two hospitals in northern Uganda. BMJ open, 2014 4(11): e005889 10.1136/bmjopen-2014-005889 25387757 PMC4244481

[7] Kebede K.M., Abateneh D.D., and Belay A.S. Hepatitis B virus infection among pregnant women in Ethiopia: a systematic review and Meta-analysis of prevalence studies. BMC infectious diseases, 2018 18(1): 322–31. 10.1186/s12879-018-3234-2 29996785 PMC6042274

[8] Ephraim R., et al Seroprevalence and risk factors of hepatitis B and hepatitis C infections among pregnant women in the Asante Akim North Municipality of the Ashanti region, Ghana; a cross sectional study. African health sciences, 2015 15(3): 709–13. 10.4314/ahs.v15i3.2 26957956 PMC4765455

[9] Anabire N.G., et al Prevalence of malaria and hepatitis B among pregnant women in Northern Ghana: Comparing RDTs with PCR. PloS one, 2019 14(2): e0210365 10.1371/journal.pone.0210365 30726218 PMC6364880

[10] Tanga A.T., et al Sero-prevalence of hepatitis B virus and associated factors among pregnant women in Gambella hospital, South Western Ethiopia: facility based cross-sectional study. BMC infectious diseases, 2019 19(1): 602–09. 10.1186/s12879-019-4220-z 31291901 PMC6617953

[11] Eyong E.M., et al The prevalence of HBsAg, knowledge and practice of hepatitis B prevention among pregnant women in the Limbe and Muyuka Health Districts of the South West region of Cameroon: a three-year retrospective study. The Pan African medical journal, 2019 32: 122–33. 10.11604/pamj.2019.32.122.16055 31312290 PMC6607245

[12] Anaedobe C.G., et al Prevalence, socio-demographic features and risk factors of Hepatitis B virus infection among pregnant women in Southwestern Nigeria. The Pan African Medical Journal, 2015 20: 406–17. https://dx.doi.org/10.11604%2Fpamj.2015.20.406.6206 10.11604/pamj.2015.20.406.6206 PMC4524914 26301010

[13] Araya Mezgebo T., et al Hepatitis B virus infection and associated risk factors among pregnant women attending antenatal care in health facilities of Tigray, Northern Ethiopia. Journal of medical virology, 2018 90(3): 503–09. 10.1002/jmv.24987 29077204

[14] Kapinga, D.R. Seroprevalence and factors associated with hepatitis b virus infection in pregnant women attending antenatal clinic in Karagwe District Council, Kagera Region. 2017, Muhimbili University of Health and Allied Sciences. http://dpsvr.muhas.ac.tz:8080/xmlui/handle/123456789/2076

[15] Gedefaw G., et al Risk factors associated with hepatitis B virus infection among pregnant women attending antenatal clinic at Felegehiwot referral hospital, Northwest Ethiopia, 2018: an institution based cross sectional study. BMC research notes, 2019 12(1): 509–16. 10.1186/s13104-019-4561-0 31416477 PMC6694615

[16] Helegbe G.K., et al Seroprevalence of Malaria and Hepatitis B Coinfection among Pregnant Women in Tamale Metropolis of Ghana: A Cross-Sectional Study. Canadian Journal of Infectious Diseases and Medical Microbiology, 2018 2018 10.1155/2018/5610981 PMC6174787 30344800

[17] Cui A.-M., et al Maternal hepatitis B virus carrier status and pregnancy outcomes: a prospective cohort study. BMC pregnancy and childbirth, 2016 16(1): 87–95. 10.1186/s12884-016-0884 27113723 PMC4845477

[18] Eke A.C., et al Prevalence, correlates and pattern of hepatitis B surface antigen in a low resource setting. Virology journal, 2011 8(1): 12–20. http://www.virologyj.com/content/8/1/12 21226907 10.1186/1743-422X-8-12 PMC3027130

[19] Esan A., et al Sero-prevalence of hepatitis B and Hepatitis C virus co-infection among pregnant women in Nigeria. Am J Biomed Res, 2014 2(1): 11–5. 10.12691/ajbr-2-1-3

[20] Day S.L., et al Prevalence, clinical and virologic outcomes of hepatitis B virus co-infection in HIV-1 positive Kenyan women on antiretroviral therapy. PLoS One, 2013 8(3): e59346 10.1371/journal.pone.0059346 23527168 PMC3601052

[21] Slowik M.K. and Jhaveri R. Hepatitis B and C viruses in infants and young children. in Seminars in pediatric infectious diseases. 2005 16 (4): 296–05. 10.1053/j.spid.2005.06.009 16210109

[22] International Alliance of Patients' Organizations. Hepatitis: A Ghana situation review. 2017 [cited 2019 December 12]; Available from: https://www.iapo.org.uk/news/2017/jul/3/hepatitis-ghana-situation-review.

[23] Ministry of Health. National Policy on Viral Hepatitis 2014, Ministry of Health Ghana.

[24] Ghana Statistical Service, Ghana Health Service, ICF International. Ghana Maternal Health Survey 2017: Key Indicators Report. 2018, Accra, Ghana: Available from: http://www2.statsghana.gov.gh/docfiles/PR95.pdf

[25] Hussein A.A., Motib A.S., and Hadi L.M. Evaluation of ELISA and HBsAg Rapid Test Cassette Assay in Detection of Hepatitis B Virus. Journal of Pharmaceutical Sciences and Research, 2018 10(12): 3157–3167.

[26] Völker F., et al Prevalence of pregnancy-relevant infections in a rural setting of Ghana. BMC pregnancy and childbirth, 2017 17(1): 172–79. 10.1186/s12884-017-1351-3 28583150 PMC5460405

[27] Ofori-Asenso R. and Agyeman A.A. Hepatitis B in Ghana: a systematic review & meta-analysis of prevalence studies (1995–2015). BMC infectious diseases, 2016 16(1): 130–45. 10.1186/s12879-016-1467-5 26987556 PMC4797341

[28] Bittaye M., et al Hepatitis B virus sero-prevalence amongst pregnant women in the Gambia. BMC infectious diseases, 2019 19(1): 259–67. 10.1186/s12879-019-3883-9 30876397 PMC6419830

